# Effects of alcohol consumption on maxillofacial fractures in simple falls

**DOI:** 10.1002/cre2.308

**Published:** 2020-07-27

**Authors:** Shunsuke Hino, Miki Yamada, Yosuke Iijima, Ryuichiro Araki, Takahiro Kaneko, Norio Horie

**Affiliations:** ^1^ Department of Oral and Maxillofacial Surgery, Saitama Medical Center Saitama Medical University Saitama Japan; ^2^ Community Health Science Center Saitama Medical University Saitama Japan

**Keywords:** maxillofacial fractures, fall, alcohol consumption

## Abstract

**Objectives:**

This study aimed to investigate the effects of alcohol consumption (AC) on maxillofacial fractures caused by falls on a level surface (simple falls).

**Material and Methods:**

Patients with maxillofacial fractures caused by falls who visited the Oral and Maxillofacial Surgery Clinic from January 2006 to December 2016 were evaluated. Patients with simple falls were subdivided into those who fell with AC (Falls with AC) and those who fell without AC (Falls without AC).

**Results:**

Of 180 patients with falls with maxillofacial fractures, 155 had simple falls, and 25 patients had falls from a height. Of the simple falls, 52 were Falls with AC, and 102 were Falls without AC. Falls with AC were significantly more frequent in males (*p* = .0005). The average number of fracture lines in the mandible was significantly higher in Falls with AC (2.13 ± 0.99 [mean ± *SD*]) than in Falls without AC (1.76 ± 0.91) (*p* = .011). The average Facial Injury Severity Scale was significantly higher in Falls with AC (3.08 ± 1.43) than in Falls without AC (2.43 ± 1.29) (*p* = .007).

**Conclusions:**

Falls with AC were associated with more severe maxillofacial fractures than Falls without AC.

## 1 INTRODUCTION

Alcohol consumption (AC) is a common habit of people, especially adult males, worldwide (Delker, Brown, & Hasin, 2016; World Health, 2007). AC and binge drinking have increased in the United States, particularly among females (Delker et al., 2016; Mokdad et al., 2018). Alcohol is one of the leading causes in the Global Burden of Disease, as it accounts for 3.9% of causes (Lim et al., 2012). Alcohol has a depressant effect on the central nervous system that reduces the cognitive ability to assess risk, reduces the ability to make rational decisions, and reduces the physical ability to escape or defend oneself (Eggensperger, Smolka, Scheidegger, Zimmermann, & Iizuka, 2007; Laverick, Patel, & Jones, 2008). At the same time, alcohol brings out aggression, resulting in interpersonal violence (Boffano et al., 2015a; Nakhgevany, LiBassi, & Esposito, 1994). There have been many reports that acute AC increases the risk of sustaining an injury (Brumback, Cao, & King, 2007; Rapoport, 2012). Furthermore, acute AC has been associated with serious injury (Watt, Purdie, Roche, & McClure, 2006).

In the oral and maxillofacial region, the effects of AC have been well documented in injuries in assaults and motor vehicle accidents (MVAs), in which AC made the injuries more severe (Hung, Montazem, & Costello, 2004; Laverick et al., 2008; Schneider et al., 2015; Soares‐Carneiro et al., 2016). However, the association between AC and the severity of injuries has rarely been reported for falls (Schneider et al., 2015). The association between falls and AC seems to be worth examining because falls are among the most frequent causes of facial bone fractures (Boffano et al., 2015b; Boffano, Kommers, Karagozoglu, & Forouzanfar, 2014). A simple fall is a fall that is not affected by height, because a fall from a height causes more severe injuries (Hino, Yamada, Araki, Kaneko, & Horie, 2019). Therefore, it is reasonable to think that a simple fall is a good example to compare the effects of AC on patients with falls. The aim of this study was to retrospectively investigate the effects of AC on maxillofacial fractures due to simple falls.

## 2 MATERIALS AND METHODS

### 2.1 Patients

This retrospective study was conducted according to the Declaration of Helsinki, with the approval of the Ethical Review Board of Saitama Medical Center.

Patients who visited the Oral and Maxillofacial Surgery Clinic, Saitama Medical Center, Saitama Medical University from January 1, 2006 to December 31, 2016 with a chief complaint of maxillofacial fracture were evaluated. Data on these patients were obtained from their clinical records and radiographs and analyzed retrospectively. To investigate the effects of AC on maxillofacial fractures in falls, patients with simple falls were identified. The patients who fell from a height were excluded, because such patients are prone to have more severe injuries whether or not they consume alcohol. In patients with simple falls, patients who were drunk when they fell constituted the Falls with AC group, and patients who were not drunk were the Falls without AC group. Whether the patients had consumed alcohol was based on the patients’ self‐reports.

Sex, age, time and place of fall, time to receiving medical attention, and severity of injuries were compared between the two groups. Time of fall was divided into six subgroups of four‐hour blocks: 0:00–<4:00, 4:00–<8:00, 8:00–<12:00, 12:00–<16:00, 16:00–<20:00, and 20:00–<24:00. The place of the fall was classified as either at home or outside the home. Outside the home included places other than the patient’s home, such as a pub, a restaurant, a house of a friend, and on the way home. Time to receiving medical attention was divided into four subgroups: <6 hr, 6–<12 hr, 12–<24 hr, and ≥24 hr.

The severity of facial injury was evaluated based on the site of fracture, number of fracture lines in the mandible, and the Facial Injury Severity Scale (FISS) (Table 1) (Bagheri et al., 2006). To investigate the general trends of injuries associated with maxillofacial fractures in falls, the frequencies of fractures of other parts of the body and of head injuries were also investigated (Bagheri et al., 2006; Shepherd, 1998).

**TABLE 1:** Facial Injury Severity Scale (FISS) (Bagheri et al., 2006)

Mandible	
Dento Alveolar	1 point
Each fracture of body/ramus/symphysis	2 points
Each fracture: condyle/coronoid	1 point
Mid‐face	
Each midfacial fracture is assigned one point, unless part of a complex	
Dento Alveolar	1 point
Le Fort I	2 points
Le Fort II	4 points
Le Fort III	6 points
(Unilateral Le Fort fractures are assigned half the numeric value)	
Naso‐Orbital Ethmoid (NOE)	3 points
Zygomatico Maxillary Complex (ZMC)	1 point
Nasal	1 point
Upper face	
Orbital roof/rim	1 point
Displaced frontal sinus/bone fractures	5 points
Non‐displaced fractures	1 point
Facial laceration	
Over 10 cm long	1 point

*Note:* The FISS is the summation of the above points in an individual patient.

### 2.2 Statistical analysis

The results were analyzed using SPSS version 20.0 for Windows (SPSS Inc, Chicago, IL). The age, sex, and place of fall of the two categories of simple falls were compared by Fisher's exact test. The time of fall and time to receiving medical attention of the two categories were analyzed by the Fisher–Freeman–Halton test. For the site of fracture, fracture lines in the mandible, and FISS, the Fisher–Freeman–Halton test and the Kruskal‐Wallis test were used to compare differences between two categories. For the average number of fracture lines in the mandible and the average FISS score, the Mann–Whitney test was used to verify possible correlations between two categories. The frequencies of fractures of other parts of the body and of head injuries were compared by Fisher's exact test. The significance level was set at 5% (*p* < .05) for all tests.

## 3 RESULTS

The total number of cases of falls was 180, with 155 (86.1%) simple falls and 25 (13.9%) falls from a height (Table 2). In simple fall patients, 53 (34.2%) were Falls with AC, and 102 (65.8%) were Falls without AC. Falls with AC were significantly more frequent in males (*p* = .001), involving persons in their third to seventh decades (Table 3). In Falls without AC, there was no significant difference between male and female patients, with no trend in the age distribution (Table 3). There was a significant difference in the time of fall between the two groups (*p* < .001), but there was no significant difference in the place of fall (*p* = .257). There was a significant difference in the time to receiving medical attention between Falls with and without AC (*p* = .021) (Table 4). With respect to site of fracture and severity of injury, most cases affected the mandible, and mid‐face fractures were relatively rare. The most frequent site of mandibular fracture in simple falls in each group was the condyle, followed by the symphysis (Table 5). For the distribution of fracture lines in the mandible and the FISS, there were no significant differences between the two groups. The average number of fracture lines in the mandible was significantly higher in Falls with AC (2.13 ± 0.99, mean ± *SD*) than in Falls without AC (1.76 ± 0.91) (*p* = .011), and the average FISS of Falls with AC was significantly higher (3.08 ± 1.43) than of Falls without AC (2.43 ± 1.29) (*p* = .007) (Table 5). There were no significant differences in the occurrence of fractures of other parts of the body and of head injuries between the two groups (Table 5).

**TABLE 2:** Classification of falls

	Male (%)	Female (%)	*N* (%)	*p* value
Simple falls	107 (69.0)	48 (31.0)	155 (100)	*p* = .251
Falls from height	14 (56.0)	11 (44.0)	25 (100)	

*Note:* Simple falls are falls on a level surface.

**TABLE 3:** Age and sex distribution of Falls without AC and Falls with AC

	Falls with AC			Falls without AC			
Age (y)	Male	Female	Total (%)	Male	Female	Total (%)
0–9	0	0	0 (0)	0	0	0 (0)
10–19	0	0	0 (0)	8	2	10 (9.8)
20–29	10	0	10 (18.9)	10	2	12 (11.8)
30–39	9	1	10 (18.9)	6	2	8 (7.8)
40–49	8	2	10 (18.9)	9	7	16 (15.7)
50–59	7	1	8 (15.1)	6	8	14 (13.8)
60–69	8	2	10 (18.9)	8	8	16 (15.7)
70–79	4	0	4 (7.54)	12	6	18 (17.6)
80–	0	1	1 (1.89)	2	6	8 (7.8)
Total	46	7	53 (100)	61	41	102 (100)

Falls with AC, patients who fell with alcohol consumption.

Falls without AC, patients who fell without alcohol consumption.

In Falls with AC, male sex was significantly more frequent (*p* = .001).

**TABLE 4:** Time and place of fall

	Falls with AC	Falls without AC	*p* value
	Total (%)	Total (%)	
Time of fall			*p* < .001
0:00–< 4:00	16 (30.1)	9 (8.8)	
4:00–< 8:00	1 (1.9)	8 (7.8)	
8:00–<12:00	1 (1.9)	27 (26.5)	
12:00–<16:00	3 (5.7)	23 (22,5)	
16:00–<20:00	5 (9.4)	15 (14.7)	
20:00–<24:00	27 (50.9)	20 (19.6)	
Total	53	102 (100)	
Place of fall			*p* = .257
At home	17 (32.1)	24 (23.4)	
Outside of home	36 (68.0)	78 (76.5)	
Total	53 (100)	102 (100)	
Time to receiving medical consultation			*p* = .021
<6 hr	32 (60.4)	80 (78.4)	
≥6 hr and <12 hr	9 (17.0)	9 (8.8)	
≥12 hr and <24 hr	3 (5.7)	0 (0.0)	
≥24 hr	9 (17.0)	13 (12.7)	
Total	53 (100)	102 (100)	

Falls with AC, patients who fell with alcohol consumption.

Falls without AC, patients who fell without alcohol consumption.

**TABLE 5:** Site of fracture and severity of injury

	Falls with AC	Falls without AC
Site of fracture		
Mandible	44	87
Midface	2	7
Mandible + midface	7	8
Total	53	102
Fracture lines in the mandible		
Alveolus	3	12
Symphysis	27	35
Body	10	16
Angle	11	9
Ramus	0	1
Condyle	55	96
Total	106	169
Average (mean ± *SD*)	2.13 ± 0.99^*^	1.76 ± 0.91
Facial Injury Severity Scale (points)		
1	8	34
2	15	23
3	5	17
4	18	24
>4	7	4
Average (mean ± *SD*)	3.08 ± 1.43^**^	2.43 ± 1.29
Fractures of other parts of the body (%)^a^	7 (13.2)	17 (16.7)
Head injuries (%)^b^	3 (5.7)	9 (8.8)

Falls with AC, patients who fell with alcohol consumption.

Falls without AC, patients who fell without alcohol consumption.

^*^ *p* = .011;

^**^ *p* = .007.

a Fractures of other parts of body include rib, clavicle, and tibial fractures.

^b^ Head injuries include hematoma and convulsion.

## 4 DISCUSSION

The objective of this study was to evaluate whether AC had an effect on the severity of maxillofacial fractures in simple falls.

The prevalence of AC in injuries differs depending on demographics, ethnicity, and patient age (Jamebozorgi, Kavoosi, Shafiee, Kahlaee, & Raei, 2013; J. J. Johnston & McGovern, 2004). Overall, alcohol‐related falls have been reported to account for 32.2% of all falls (Jamebozorgi et al., 2013). The presence of AC in maxillofacial injury has been reported to range from 11.0 to 47.0% (Laverick et al., 2008; Weihsin et al., 2014). In detail, AC has been reported to be involved in 12.0 to 23.0%, 7.0 to 72.0%, and 11.0 to 24.0% of MVAs, assaults, and falls, respectively (Boffano et al., 2015a; Laverick et al., 2008; Weihsin et al., 2014). Limited to patients with maxillofacial fractures, AC has been found to be involved in 10.9% of MVAs, 23.0 to 70.8% of assaults, and 9.7% of falls (Eggensperger et al., 2007; Schneider et al., 2015). In the present report of patients with maxillofacial fractures due to falls, 34.2% of patients had associated AC, higher than in previous reports. This may be because Falls with AC in the present study strictly included all patients who had consumed alcohol, even though they had drunk a minimal amount and were sober, because an ethnic difference in alcohol metabolism has been suggested. Injuries associated with AC have been reported to be higher in men than in women (Ammendola et al., 2000; Delker et al., 2016). This has been explained by the fact that women infrequently consume alcohol, drink less frequently outside of the home, and have biologically and physiologically different alcohol metabolism than men (Rha, Kim, Han, Park, & Yoo, 2017). Male predominance in Falls with AC in the present study was consistent with previous reports. Falls with AC occurred predominantly in the second to seventh decades, but Falls without AC were constant from teenagers to elderly persons. AC may be an important factor in falls of every drinker, irrespective of age (Nevitt, Cummings, & Hudes, 1991; Roccia, Boffano, Bianchi, & Zavattero, 2014). Falls with AC had a trend to occur more often in the evening and middle of the night, and this was because opportunities for alcohol intake are clearly more frequent in that time period. Falls outside of the home were significantly more frequent than those at home in both Falls with AC and Falls without AC, but there was no significant difference in the place of falls between Falls with AC and Falls without AC. It was suggested that one must be careful about falls when outside of the home, both with and without AC. Time to receiving medical attention was found to be prolonged in Falls with AC. Some patients in the Falls with AC group in the present study stated that they had quickly gone to sleep after returning home without awareness of having fallen. In Falls with AC, the delay in noticing the injury might delay receiving medical attention.

It is difficult to accurately determine the amount of AC, because patients' self‐reports of AC are usually less than the amount that they actually consumed (Honkanen & Smith, 1990). To discuss the physiological effects of alcohol, blood alcohol concentrations (BACs) need to be measured. BAC has a close association with the effects of alcohol (Harrison et al., 2017). The effect of alcohol varies from person to person depending on metabolism, such as alcohol dehydrogenase activity, but a common guideline for the effect of alcohol is as follows: very low BACs (<100 mg/L) produce zero or undetectable effects; BAC of 300 mg/L produces impairment of motor skills; BAC of 1.5 g/L produces gross motor impairment; and BAC of 2 g/L produces amnesia and coma (Harrison et al., 2017; Jamebozorgi et al., 2013). Clinically, BAC of over 1 g/L causes significant swaying and decreased attention, visual acuity, and adaptation to brightness and glare (Hingson & Howland, 1993). Regarding injuries to the maxillofacial region, the majority of patients involved in MVAs had a BAC > 1 g/L (Nakhgevany et al., 1994).

Patients with AC have been reported to be more likely to be injured (Rapoport, 2012; Vinson, Maclure, Reidinger, & Smith, 2003), and patients with AC have been reported to have more severe injuries than patients without AC (Jamebozorgi et al., 2013; Kowalenko, Burgess, Szpunar, & Irvin‐Babcock, 2013; Roccia et al., 2014). As to the dose of alcohol consumed, it has been commonly reported that there is a positive correlation between BAC and severity of injury (Jamebozorgi et al., 2013; Ronning et al., 2015).

In maxillofacial injuries, AC has a close association with MVAs and assaults (Soares‐Carneiro et al., 2016). AC with MVAs and assaults was frequent in males, especially young males, and AC has been shown to significantly increase the severity of maxillofacial injuries (Eggensperger et al., 2007; O’Meara, Witherspoon, Hapangama, & Hyam, 2011; Soares‐Carneiro et al., 2016). In sports injuries, among the leading causes of maxillofacial injuries, AC has not been frequently associated with maxillofacial injuries (Cavalcanti‐Galdino, Silva, Mendes, Santos, & Simas, 2014; Laverick et al., 2008). Although AC is associated with more severe injury in common falls, not many reports have investigated the association between AC and the severity of maxillofacial injuries due to falls. In the present study, since the average number of fracture lines in the mandible and the FISS were significantly higher in Falls with AC, AC appeared to be the risk factor for more severe maxillofacial injuries, as for other injuries caused by falls.

It has been reported that differences in BAC cause differences in injuries, and the higher the BAC, the higher was the frequency of cerebral injuries, and the lower was the frequency of limb injuries (Nakhgevany et al., 1994). However, in the present study, there was no significant difference in fractures affecting other parts of the body and in cerebral injuries between Falls with AC and Falls without AC. Further examination of BAC will be needed to elucidate the association between AC and severity and comorbidity of maxillofacial injuries caused by falls.

Alcohol consumption has an effect on the central nervous system, which includes ocular effects (Cavalcanti‐Galdino et al., 2014). There have been many studies showing that alcohol intake impairs visual and oculomotor functions (Brasil et al., 2015; Cavalcanti‐Galdino et al., 2014). In association with MVAs, the impairment is thought to be important in impeding safe driving, and some suggested that the impairment was caused even with low BACs (K. Johnston, Timney, & Goodale, 2013; Watten & Lie, 1996). Since Falls with AC were more frequent in the evening to night time with poor visibility, an ocular disturbance might be one of the reasons for Falls with AC.

There were some limitations in the statistical analysis of the present study, including the relatively low number of participants, especially with Falls with AC. Second, BAC was not examined. Further studies with BAC measurement will offer more information for the association between the severity of maxillofacial fractures in simple falls and AC.

## 5 CONCLUSION

Maxillofacial fractures in falls with AC occurred over a wide range of ages in males. Falls with AC appear to be associated with more severe maxillofacial fractures than Falls without AC.

## CONFLICT OF INTEREST

The authors confirm that they have no conflicts of interest.

## ETHICS STATEMENT

The Ethics Review Board at Saitama Medical University approved the protocol of the study, which proceeded in accordance with the Declaration of Helsinki.
